# Insights into oncovirus-driven tumor dynamics: a perspective from intravital imaging

**DOI:** 10.3389/fcell.2026.1830182

**Published:** 2026-06-04

**Authors:** Hyeri Kim, Dohun Pyeon, Sangbum Park

**Affiliations:** 1 Institute for Quantitative Health Science and Engineering, Michigan State University, East Lansing, MI, United States; 2 Department of Microbiology, Genetics, and Immunology, Michigan State University, East Lansing, MI, United States; 3 Department of Medicine, Michigan State University, East Lansing, MI, United States; 4 Department of Pharmacology and Toxicology, Michigan State University, East Lansing, MI, United States

**Keywords:** immune cell infiltration, intravital imaging, multiphoton microscopy, oncovirus, tumor microenvironment, HPV, head and neck cancer

## Abstract

The tumor microenvironment (TME) is a critical determinant of cancer initiation, progression, and therapeutic response. It comprises not only tumor cells but also immune cells, stromal components, and vasculature that interact through complex molecular signaling networks. In cancers driven by oncoviruses, viral infection represents a unique biological factor that profoundly influences TME formation and remodeling, thereby shaping distinct features such as immune evasion, chronic inflammation, and stromal and vascular reprogramming. Conventional approaches provide valuable insights into the static composition of the TME, but are inherently limited in capturing dynamic cellular behaviors, including immune cell migration, spatiotemporal immune remodeling, and CD8^+^ T cell interactions with virally infected tumor cells over time, which are critical for understanding immune evasion and therapeutic responses in oncovirus-associated cancers. In this review, we briefly summarize virus-associated cancers and the mechanisms by which oncoviruses actively shape and regulate the TME. We further highlight the importance of intravital imaging as a powerful approach for directly visualizing the spatial and temporal dynamics of the TME in live animals and discuss how this technology can advance our understanding of oncovirus-driven TME remodeling and the development of effective anticancer therapeutic strategies.

## 1 Introduction

The tumor microenvironment (TME) is a complex and dynamic ecosystem composed of cancer cells and diverse immune and stromal cell populations, including cancer-associated fibroblasts (CAFs), tumor-associated macrophages (TAMs), cytotoxic CD8^+^ T lymphocytes (CTLs), regulatory T cells (Tregs), myeloid-derived suppressor cells (MDSCs), mesenchymal stem cells (MSCs), extracellular matrix (ECM), vascular endothelial cells, adipocytes, and neuroendocrine (NE) cells (Salo et al., 2014; Xiao et al., 2025; Wei et al., 2020). Beyond tumor-intrinsic genetic alterations, the surrounding microenvironment plays key roles in cancer initiation, progression, invasion, metastasis, angiogenesis, immune evasion, tumor-promoting inflammation, and therapeutic response (Salo et al., 2014; Bilotta et al., 2022; Wang et al., 2017; Khan and Malik, 2024). In particular, the spatial distribution and functional state of immune cells within the TME have emerged as key indicators closely associated with prognosis and treatment outcomes.

Approximately 15%–20% of human cancers are estimated to be associated with viral infection (Contreras et al., 2024; Nam et al., 2025). Major oncoviruses include human papillomavirus (HPV), Epstein-Barr virus (EBV), hepatitis B virus (HBV), hepatitis C virus (HCV), human T cell leukemia virus type 1 (HTLV-1), Merkel cell polyomavirus (MCPyV), and Kaposi’s sarcoma-associated herpesvirus (KSHV) (Schiller et al., 2021). While infecting distinct tissues and cell types, these oncoviruses often establish chronic infections and share the common features of persistent interactions with the host immune system (Zheng, 2010; Chen et al., 2014). Viral antigens initially stimulate antiviral immunity, but their persistent presence may progressively promote immune dysfunction, including CD8^+^ T cell exhaustion, enrichment of immunosuppressive myeloid cell populations, and sustained secretion of inhibitory cytokines (Pulliam et al., 2024; Sang et al., 2025; Wu et al., 2018; Choi and James Ou, 2006; Chen et al., 2015; Harhaj and Shembade, 2020). In contrast to many non-viral tumors, in which antigenic landscapes are often heterogeneous and evolve dynamically as a consequence of accumulated somatic mutations, oncovirus-associated cancers often exhibit persistent expression of viral antigens that can provide a sustained and relatively defined source of immune stimulation. Although these tumors may also display substantial heterogeneity and present non-viral antigens, chronic antigen exposure may promote temporally prolonged and spatially organized immune remodeling within the TME. Accordingly, oncovirus-associated cancers offer a useful framework for studying how chronic antigenic stimulation and inflammation reshape the TME over time.

Unlike static endpoint analyses, understanding the progression from viral infection to tumorigenesis requires approaches capable of capturing the spatial and temporal evolution of immune-tumor interactions within intact living tissues. Because oncovirus-associated tumorigenesis involves continuous immune surveillance, chronic inflammation, and progressive microenvironmental remodeling, intravital imaging approaches are particularly well suited for investigating these processes.

A substantial portion of conventional virus and TME research has relied on *in vitro* systems. These systems are effective for elucidating molecular mechanisms, including viral entry, intracellular trafficking, replication, release, and protein interactions (Weber et al., 2023; Giehler et al., 2024; Yang et al., 2023; Cui et al., 2023). However, they remain limited in recapitulating tissue-specific cellular composition, spatial organization between cells, immune cell motility, blood or lymphatic flow, and the physical properties of the ECM. The progression from viral infection to cancer formation is not merely an intracellular event but rather a spatiotemporally coordinated process involving localized infection, immune cell recruitment, and progressive remodeling of the microenvironment. To address these limitations, intravital imaging has been used to visualize cellular and subcellular dynamics within intact living tissues in real time (Pittet and Weissleder, 2011; Ellenbroek and van Rheenen, 2014; Beerling et al., 2011). This approach allows quantitative analysis of immune cell migration, motility, arrest, and dwell time, reflecting the duration of immune cell retention and interaction within specific tissue regions, as well as the duration of physical cell–cell interactions *in vivo* (Pittet and Weissleder, 2011; Hirao et al., 2025). As such, intravital imaging provides a powerful platform for visualizing dynamics of the TME remodeling in living organisms, offering the potential to track and quantify the spatiotemporal progression of virus-associated tumorigenesis (Zomer et al., 2011; Harney et al., 2015; Cools Lartigue et al., 2013; Honda et al., 2023). Furthermore, intravital imaging can be applied to investigate cellular responses to diverse therapeutic strategies in virus-associated cancers (Yang et al., 2025). Therefore, broader application of this approach may provide critical insights into the dynamic mechanisms underlying immune evasion and tumor progression in virus-associated tumors.

In this mini-review, we discuss how intravital imaging can provide unique insight into the spatiotemporal evolution of oncovirus-driven TMEs and highlight its potential applications in understanding dynamic immune-tumor interactions and developing future therapeutic strategies.

## 2 Oncovirus-driven remodeling of the TME

### 2.1 Oncoviruses and associated cancers

Oncoviruses give rise to a wide spectrum of malignancies depending on their tissue tropism, viral life cycles, and pathogenic mechanisms (Schiller et al., 2021). The seven major oncoviruses, including HPV, EBV, HBV, HCV, HTLV-1, KSHV, and MCPyV, are causally linked to human cancers. These viruses are associated with diverse tumors, including cervical cancer, head and neck squamous cell carcinoma, hepatocellular carcinoma, lymphomas, Kaposi’s sarcoma, and Merkel cell carcinoma (Hsu et al., 2015; Dispenzieri and Fajgenbaum, 2020; Broussard and Damania, 2020; Calvaruso et al., 2018; Yete et al., 2017; Kerr, 2019).

These oncoviruses differ in genome types and replication strategies. While HPV and MCPyV encode viral oncoproteins that directly target host tumor suppressors, HBV and HCV drive tumorigenesis primarily through chronic inflammation, despite their different genome types (DNA and RNA, respectively) (Bouchard and Navas-Martin, 2011; Gaglia and Munger, 2018). EBV and KSHV, as large DNA viruses that encode several oncogenes, establish latent infections and cause chronic inflammation (Cesarman, 2014; Young and Murray, 2003; Uppal et al., 2014). Despite these biological differences, all major oncoviruses establish persistent infections. Acute infection alone is generally insufficient to induce malignant transformation; instead, tumor development typically arises over years to decades through sustained host–virus interaction and incomplete immune clearance.

Oncoviruses employ several convergent molecular mechanisms to promote oncogenesis. Viral oncoproteins such as HPV E6 and E7, EBV Latent Membrane Protein 1 (LMP1) and Epstein-Barr Nuclear Antigen 1 (EBNA1), HBV Hepatitis B virus X protein (HBx), HTLV-1 Trans-Activator X protein (Tax) and HTLV-1 Basic Leucine Zipper Factor (HBZ), and MCPyV large and small T antigens disrupt tumor suppressors, including p53 and retinoblastoma (pRB), leading to dysregulated cell-cycle progression and impaired apoptosis (Frappier, 2012; Gao and Zheng, 2011; Bhattacharjee et al., 2022; Sivasudhan et al., 2022; El Sharkawy et al., 2018). Persistent infection contributes to the accumulation of genomic instability by suppressing DNA damage responses and, in some viruses such as HTLV-1, HBV, HPV, and MCPyV, by direct viral integration into host chromosomes (Jiang et al., 2021; Ahmed et al., 2022; Lemoine and Marriott, 2002). Several oncoviruses further enhance tumor progression by promoting angiogenesis and metastasis. Viral proteins such as EBV LMP1, KSHV G protein-coupled receptor (vGPCR), and HBV HBx induce angiogenic factors, including vascular endothelial growth factor (VEGF) and angiopoietins, resulting in abnormal vascularization that facilitates tumor cell survival and invasion, immune suppression, and metastatic dissemination (Wang and Ning, 2021; Mesri et al., 2013; Elpek, 2021; Rivera Soto and Damania, 2019). An overview of major human oncoviruses, including their genome types, associated cancers, and oncogenic mechanisms, is summarized in Table 1.

**TABLE 1:** Major human oncoviruses and their oncogenic mechanisms.

Oncovirus	Virus family (genome type)	Associated cancers	Dominant oncogenic mechanisms	Feasibility for intravital imaging
HPV	Papillomavirus (dsDNA)	Cervical cancer, HNSCC anogenital cancers	E6-mediated p53 degradation, E7-mediated Rb inactivation, and genomic instability	High; MmuPV1 infection models and HPV-associated murine models (e.g., mEERL)
EBV	Herpesvirus (dsDNA)	Nasopharyngeal carcinoma, gastric cancer, lymphomas	Latent oncogenes, NF-κB and JAK–STAT signaling	Moderate; humanized mouse models available
HBV	Hepadnavirus (gapped dsDNA)	Hepatocellular carcinoma	HBx signaling, chronic inflammation	Moderate; liver-humanized mouse models available
HCV	Flavivirus (ssRNA)	Hepatocellular carcinoma	Chronic inflammation, oxidative stress	Moderate; liver-humanized mouse models available
HTLV-1	Retrovirus (ssRNA)	Adult T cell leukemia, lymphoma	Tax/HBZ-driven genomic instability	Low; limited models
KSHV	Herpesvirus (dsDNA)	Kaposi’s sarcoma	Latent oncogenes, NF-κB and JAK–STAT signaling	Low; technically challenging humanized models
MCPyV	Polyomavirus (dsDNA)	Merkel cell carcinoma	Large and small T antigens	Low; limited animal models

dsDNA, double-stranded DNA; ssRNA, single-stranded RNA.

Feasibility was qualitatively assessed based on the availability of relevant animal models, tissue accessibility, and compatibility with longitudinal intravital imaging.

### 2.2 The roles of oncoviruses in remodeling the TME

**FIGURE 1:**
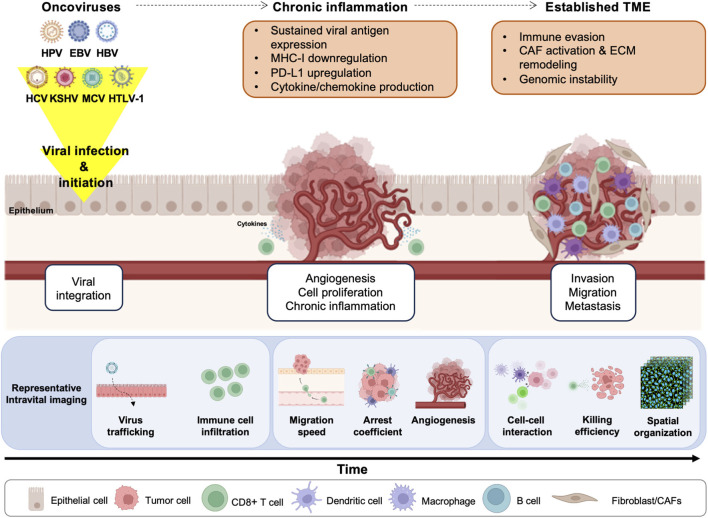
Schematic illustration of tumor microenvironment (TME) formation during oncovirus-driven cancer progression. Oncovirus-driven tumorigenesis begins with infection of susceptible host cells and integration or persistence of the viral genome. Sustained viral antigen expression and chronic inflammatory signaling promote cytokine production, angiogenesis, and aberrant cell proliferation. Over time, these processes progressively remodel the tissue architecture, leading to the establishment of a complex TME characterized by stromal activation, immune cell infiltration, spatial heterogeneity, and invasive behavior. Representative intravital imaging-derived readouts that can be used to interrogate these dynamic processes include viral trafficking, immune cell infiltration, migration speed, arrest behavior, angiogenesis, cell-cell interactions, cytotoxic activity, and spatial organization of immune and stromal compartments. Together, this schematic illustrates how persistent virus-host interactions drive the temporal evolution of an immunologically altered and spatially organized TME in oncovirus-associated cancers. Figure created with https://BioRender.com.

After tumor initiation, oncoviruses function not only as drivers of carcinogenesis but also as key regulators that influence the evolutionary progression of the TME. These processes collectively promote immune evasion, chronic inflammation, and reprogramming of stromal and vascular systems (Blaylock, 2019) (Figure 1). Oncoviruses maintain long-term interactions with the host immune system. Viral antigen expression initially elicits antiviral immune responses. However, persistent antigen expression gradually drives T cell exhaustion, upregulation of immune checkpoint molecules, and expansion of immunosuppressive cells, such as Tregs and MDSCs (Xiao et al., 2025; Aghamajidi et al., 2022). During tumor progression, immunoediting selectively eliminates highly immunogenic tumor cells, allowing immune-evasive variants to persist and expand in the TME. This process progressively establishes an immunosuppressive TME. Compared with generally heterogeneous non-viral tumors with diverse neoantigen landscapes, oncovirus-associated cancers are characterized by the persistent expression of viral antigens, providing a sustained, relatively defined source of immune stimulation. As a result, these virus-associated tumors may exhibit more temporally prolonged and spatially structured immune responses, including persistent T cell activation, immune exhaustion, and immunosuppressive niches within the TME. Viral oncoproteins play critical roles in remodeling the TME. For example, HPV E6/E7 and EBV latent proteins inhibit antigen presentation and interferon (IFN) signaling, and HTLV-1 Tax dysregulates IFN signaling (Contreras et al., 2024; Schiller et al., 2021; Boxus and Willems, 2009). HBV and HCV promote persistent hepatic inflammation that drives sustained immune activation and repeated cycles of hepatocyte injury and compensatory regeneration (turnover), creating a chronic pro-fibrotic, pro-mutagenic milieu that supports hepatocarcinogenesis (Contreras et al., 2024; Choi and James Ou, 2006). Various viral mechanisms converge to produce immune suppression and evasion across virally associated malignancies.

Repeated cycles of tissue injury and repair during persistent viral infection lead to sustained activation of nuclear factor-κB (NF-κB), signal transducer and activator of transcription (STAT3), and hypoxia-inducible factor 1α (HIF-1α) signaling. These signaling pathways promote chronic inflammation by producing pro-inflammatory cytokines (IL-1β, IL-6, and TNF-α) and reactive oxygen and nitrogen species (Boxus and Willems, 2009; Zhang et al., 2017; Digifico et al., 2021). Furthermore, this inflammatory milieu contributes to DNA damage, fibroblast activation, extracellular matrix remodeling, and the establishment of tumor-promoting stromal niches. The TME may appear immunologically active yet functionally ineffective, characterized by chronic inflammation coupled with impaired cytotoxic immunity. For example, HPV-associated head and neck squamous cell carcinoma often exhibits substantial CD8^+^ T cell infiltration. However, persistent antigen exposure and multiple immune evasion mechanisms can induce T cell exhaustion and impaired cytotoxic functionality, ultimately resulting in ineffective anti-tumor immune responses (Elmusrati et al., 2021).

In addition, oncovirus-associated cancers frequently exhibit abnormal vascular remodeling. Viral proteins, such as EBV LMP1, KSHV vGPCR, and HBV HBx, induce angiogenic factors, including VEGF and angiopoietins (Wang and Ning, 2021; Mesri et al., 2013; Elpek, 2021; Rivera Soto and Damania, 2019). This leads to increased microvessel density, aberrant capillary branching, hyperpermeable vasculature, and heterogeneous perfusion. Functionally, these vascular abnormalities enhance nutrient delivery to support tumor growth while also elevating interstitial pressure, impairing efficient drug penetration, and promoting perivascular immune cell trapping. As a result, immune cells may accumulate near blood vessels while remaining excluded from tumor cores, thereby generating pronounced spatial heterogeneity within the TME. Regions densely infiltrated by immune cells can coexist with immune-desert areas, directly influencing tumor progression and therapeutic responses. Collectively, these general mechanisms illustrate how persistent viral infection drives progressive remodeling of immune, stromal, and vascular compartments within the TME.

## 3 Intravital imaging of oncovirus-driven TME and immunotherapy dynamics

### 3.1 Intravital imaging of oncovirus-driven TME

**FIGURE 2:**
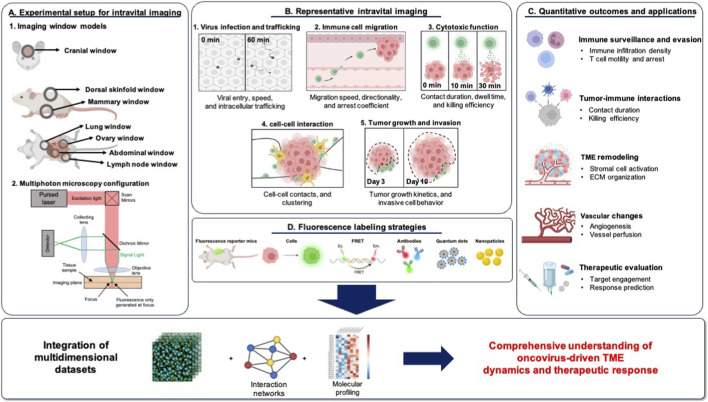
Experimental framework and multidimensional analyses of oncovirus-driven tumors using intravital imaging. **(A)** Experimental setup for intravital imaging, including imaging window models and mutiphoton microscopy configuration. **(B)** Representative intravital imaging applications, including visualization of viral infection and trafficking, immune cell migration, cytotoxic function, cell-cell interactions, and tumor growth and invasion. **(C)** Quantitative outcomes and applications, including immune-surveillance and evasion, tumor-immune interactions, TME remodeling, vascular changes, and therapeutic evaluation. **(D)** Fluorescence labeling strategies used for simultaneous tracking of viral, immune, stromal, and tumor compartments *in vivo*. Intravital imaging enables real-time visualization of dynamic cellular processes within living oncovirus-driven tumors. Shown are representative experimental setups, including window-based imaging models (e.g., cranial, dorsal skinfold, mammary, lung, and abdominal imaging windows) and a multiphoton microscopy configuration. Representative applications include visualization of viral infection and trafficking, immune cell infiltration and migration, cytotoxic interactions, and tumor growth and invasion. Fluorescence labeling strategies allow simultaneous tracking of viral, immune, stromal, and tumor compartments *in vivo*. Quantifiable outcomes include immune surveillance and evasion, tumor-immune interactions, TME remodeling, vascular changes, and therapeutic evaluation. Integration of these spatiotemporal datasets with interaction network analysis and molecular profiling provides a comprehensive mechanistic understanding of oncovirus-driven TME dynamics and therapeutic responses. Figure created with https://BioRender.com.

Tumor cells and surrounding immune and stromal components exhibit dynamic changes in abundance, spatial organization, and functional states during tumor progression and can differ markedly across distinct microanatomic regions within the same tumor (e.g., core vs. invasive margin vs. perivascular niches). Interpreting such complexity as a single static state fails to capture the evolving nature of tumor–immune–stromal interactions, because TME remodeling is inherently dynamic. Spatiotemporal information is particularly critical in oncovirus-driven cancers, where immune evasion is progressively reinforced through repeated, time-dependent cellular interactions rather than being established instantaneously (Li et al., 2025) (Figure 2). A comprehensive understanding of the TME, therefore, depends on resolving when and where immune-tumor interactions occur, how long they persist, and how their qualitative features change over time. Conventional *in vitro* systems cannot fully recapitulate the cellular complexity and spatial organization of tissues, highlighting the importance of *in vivo* imaging approaches (Sewald, 2018). Intravital imaging uniquely provides *in vivo*, spatiotemporal resolution of tumor–immune interactions, revealing mechanisms of TME remodeling and immune function that endpoint snapshots cannot capture or reliably infer.

To elucidate the spatiotemporal dynamics of oncovirus entry and TME formation, several studies have applied intravital *in vivo* imaging (Contreras et al., 2024; Sewald et al., 2015; Lertworapreecha et al., 2009). A representative example investigated how retroviruses disseminate within the secondary lymphoid organs of living animals. In detail, fluorescently labeled MLV particles were subcutaneously injected into the footpad of anesthetized mice, and their arrival at the draining popliteal lymph node (pLN) was monitored using intravital imaging (Sewald et al., 2015). These studies demonstrated that incoming MLV particles were predominantly captured by CD169^+^ CD11b^+^ macrophages (∼80%) at the subcapsular sinus. Within the pLN, viral particles were exclusively associated with CD169^+^ macrophages. Rather than eliminating the virus, CD169^+^ macrophages formed prolonged synaptic contacts with permissive B-1 cells and facilitated trans-infection, enabling viral spread deeper into lymphoid tissue. Two-photon microscopy enabled real-time visualization of these interactions, demonstrating that viral dissemination is not random but is spatially organized and dependent on defined cellular interfaces within the tissue microarchitecture.

Additionally, in an HBV pathogenesis mouse model, hepatic homing of CD8^+^ effector T cells (CD8 TE) was shown to be independent of selectins, β2-and α4-integrins, Platelet Endothelial Cell Adhesion Molecule-1 (PECAM-1), Vascular Adhesion Protein-1 (VAP-1), Guanine nucleotide-binding protein G(i) subunit alpha (Gαi)-coupled chemokine receptors, or even antigen recognition (Guidotti et al., 2015). CD8 TE exhibited crawling behavior along liver sinusoids at an average speed of approximately 10 μm/min (compared to sinusoidal blood flow of 100–400 μm/s). Upon approaching hepatocytes expressing cognate antigen, CD8 TE reduced their migration speed and ultimately arrested. In contrast, CD8 TE that did not encounter antigen continued bidirectional crawling within the sinusoids. These findings highlight the link between T cell motility and antigen recognition *in vivo*. Furthermore, intravital imaging clarified previously unresolved molecular and spatiotemporal mechanisms underlying platelet-mediated CD8 TE accumulation in the liver, providing important insights into HBV and HCV pathogenesis. It suggests that similar mechanisms can operate in infections targeting hepatocytes, including bacterial and parasitic diseases. Finally, intravital imaging has been applied to HPV-associated tumor models. In a dorsal skin-fold window chamber model, HeLa cervical cancer cells were implanted, and tumor microvascular architecture was monitored longitudinally (Lertworapreecha et al., 2009). Tumor size increased proportionally with the number of implanted cells, and tumor growth rates increased linearly. Intravital imaging revealed increased neocapillary density at the implantation site within 2 weeks, demonstrating that angiogenesis occurs early during tumor development. These findings indicate that tumor progression is not merely uniform cellular expansion but rather a temporally ordered and spatially structured process of microenvironmental remodeling. Subsequent development of animal papillomavirus models, including *Mus musculus* papillomavirus type 1 (MmuPV1), has further enabled real-time investigation of TME formation and immune evasion strategies (Uberoi et al., 2018).

Collectively, these studies demonstrate that intravital imaging, combined with oncovirus pathogenesis models and window chamber systems, has enabled real-time elucidation of viral dissemination, immune regulation, and TME formation. Intravital imaging approaches reveal that virus-mediated cancer is not simply a cell-intrinsic transformation event but a dynamically reconstructed tissue-level process.

### 3.2 Spatial and dynamic determinants of immunotherapy in oncovirus-driven cancers

Current immunotherapeutic approaches include oncolytic virotherapy, immune checkpoint inhibitors, cancer vaccines, and Chimeric Antigen Receptor T cell (CAR-T) cell therapy (Zhang and Zhang, 2020). Virus-associated cancers have occasionally demonstrated greater responsiveness to immunotherapy than non-viral tumors (Li and Zhang, 2021; Julian et al., 2021; Zhou et al., 2023; Bradford et al., 2020). However, oncovirus-driven cancers exhibit distinct immunological profiles, suggesting that their responses to immunotherapy may differ from those of non-viral tumors. Notably, low tumor mutational burden and elevated Programmed cell death protein 1 (PD-L1) expression are frequently reported in virus-associated cancers (Nam et al., 2025; Wong et al., 2015; Cancer Genome Atlas Network, 2015). Elucidating the mechanisms underlying immunotherapy responses, therefore, remains a critical task. Intravital imaging is a powerful platform for investigating immune cell dynamics during cancer immunotherapy and for uncovering novel mechanisms across diverse therapeutic strategies (Yang et al., 2025; Deng et al., 2024). Although the studies discussed in this section were not conducted exclusively in *bona fide* oncovirus-driven tumor models, they illustrate broadly applicable intravital imaging methodologies that can be translated to oncovirus-associated cancers to investigate immune cell function and therapeutic responses *in vivo*.

Several studies have applied intravital imaging to dissect the mechanisms of current immunotherapies. Fluorescence resonance energy transfer (FRET)-based intravital imaging was employed to quantitatively evaluate the efficiency and dynamics of CTL-mediated tumor killing within the liver tumor microenvironment and to investigate how the immunotolerant environment of the liver influences CTL-mediated tumor killing (Liu et al., 2021). This study established a multicolor intravital imaging platform applicable to the liver TME. CTL movement was tracked, and CTL-tumor cell interactions were assessed using a liver metastasis model. Compared with ovalbumin (OVA)-negative tumors, CTLs in OVA-positive lesions exhibited significantly reduced migration distances and decreased mean velocities upon antigen recognition. Furthermore, antigen-engaged CTLs spent the majority of their time in an arrested state. Importantly, this reduced motility reflects antigen recognition and stable immune synapse formation rather than impaired T cell function. These findings suggest that limited CTL migration within hepatic metastases represents productive immune-tumor engagement and provides functional insight into organ-specific constraints on CTL-mediated tumor killing.

Intravital imaging was also utilized to evaluate the therapeutic efficacy of Rapid Antiangiogenesis Mediated By Oncolytic virus (RAMBO), an oncolytic herpes simplex virus-1 (oHSV) engineered to express vasculostatin (Vstat120), in highly vascularized malignant soft tissue sarcoma (STS) (Nair et al., 2020). Efficient viral infection and intratumoral spread were clearly observed following RAMBO administration. At 6 days post-infection, tumor cells adjacent to vasculature had eliminated viral infection, and non-infected tumor regions exhibited regrowth. These findings indicate that viral replication and dissemination may be constrained by innate antiviral immune responses. However, early localized tumor cell death induced by viral infection may reverse the immunosuppressive TME, promote the release of tumor-associated antigens, and facilitate cross-presentation and the recruitment of antitumor T cells. Thus, precise modulation of the early innate immune response triggered by RAMBO appears critical for maximizing therapeutic efficacy. Future studies employing immunocompetent tumor models will be necessary to evaluate the impact of RAMBO on immune cell recruitment within the TME.

In addition, intravital imaging has been used to validate an enhanced adoptive cell therapy (ACT) strategy for hepatocellular carcinoma (HCC) that employs “super” NK cells engineered with specific, universal, and permeable properties via aptamer conjugation (Zhang et al., 2020). Specifically, an HCC-targeting aptamer (TLS11a) and a PD-L1-blocking aptamer were conjugated either individually or in combination to NK cells. Stereoscopic fluorescence microscopy-based intravital imaging demonstrated that NK cells conjugated with both TLS11a and PD-L1-blocking aptamers penetrated deeply into tumor tissues. These findings suggest that aptamer-equipped NK cells possess considerable potential in clinical adoptive cell immunotherapy.

Finally, a multifocal HCC model was generated using hydrodynamic gene transfer, which genetically modified the cells to activate the *c-myc* oncogene, disable the *p53* tumor suppressor, and express tracking markers and immune targets (EGFP, luciferase, and the human gp100 antigen). This model was used to evaluate immunotherapeutic approaches, including immune checkpoint inhibitors, cytokines, and adoptive T cell therapies, while mechanistic interrogation was performed through intravital imaging (Ochoa et al., 2023). A triple immunomodulatory regimen (anti-PD-1, anti-CTLA-4, and IL-2) demonstrated T cell–dependent antitumor efficacy, resulting in marked T cell infiltration. By combining resting T cell transfer with triple immunomodulation, tumor-specific T cells were tracked *in vivo* using intravital imaging. Endogenous T cells were observed infiltrating both tumor tissue and surrounding parenchyma, with particularly prominent accumulation in perivascular regions. Moreover, following immunomodulatory therapy, enhanced T cell mobility and directional migration were observed within tumors. These behavioral changes were dependent on immune checkpoint blockade combined with IL-2 treatment and were accompanied by indirect evidence of disruption of EGFP-positive tumor cells after therapy.

Collectively, therapeutic responses in oncovirus-driven cancers cannot be adequately explained by static parameters alone, such as immune cell abundance or tumor size. Instead, treatment outcomes are governed by spatial and temporal factors, including immune cell positioning, motility, duration of cellular interactions, antibody distribution, and sequestration within the TME. Intravital imaging enables real-time visualization of these dynamic processes and has emerged as a powerful tool for directly identifying the cellular events underlying therapeutic success or failure. Future application of these methodologies to physiologically relevant oncovirus-driven tumor models will enable direct visualization of how immunotherapies modulate immune-tumor interactions and overcome chronic antigen-driven immune dysfunction. Moving forward, this technology will serve as a critical platform for the design of rational combination therapy, the elucidation of resistance mechanisms, and the development of predictive biomarkers in oncovirus-associated malignancies.

## 4 Current limitations and challenges in oncovirus-focused intravital imaging

As highlighted by the studies discussed above, the application of intravital imaging to investigate oncovirus-driven TME formation and responses to therapies remains limited. In particular, relatively few studies have employed intravital imaging in *bona fide* oncovirus-driven tumor models that faithfully recapitulate natural viral infection, persistent virus-host interactions, and stepwise tumor development *in vivo*. A primary reason is the lack of appropriate animal models that faithfully recapitulate authentic viral infection and oncovirus-associated tumorigenesis. Since many human oncoviruses, including HPV, EBV, KSHV, and MCPyV, exhibit strong species specificity, current mouse models cannot recapitulate the process from natural virus infection to TME formation and cancer progression. For HBV and HCV, studies often rely on hepatocyte-derived tumor models based on established HCC cell lines rather than infection-driven tumor models (Rougier et al., 2007; Nakagawa et al., 1997; Imadome et al., 2011; Caduff et al., 2021). Additionally, generating suitable transgenic, humanized, or xenograft mouse models is time-consuming, and even when established, these models often fail to fully reproduce viral infection dynamics, life cycle progression, and carcinogenesis. Consequently, many studies focus primarily on therapeutic response testing rather than comprehensive modeling of infection-driven TME evolution. Encouragingly, the recent development of mouse oncovirus models, including MmuPV1-based infection models and HPV-positive murine syngeneic tumor models such as mEERL, provides new opportunities to investigate the TME and therapeutic strategies in live animals using intravital imaging (Uberoi et al., 2018; Lucido et al., 2014).

Intravital imaging requires technical preparation to expose tissues in live animals, ranging from simple stabilization to complex microsurgery (Looney et al., 2011). These procedures cause tissue perturbation depending on location and skill, necessitating immobilization, support, and careful hydration and temperature control for stable, longitudinal imaging. Such manipulations might trigger immune responses (Ritsma et al., 2013), limiting imaging duration. To address this, optimized imaging windows and stabilization systems help minimize immune effects and variability (Alieva et al., 2014; Vinegoni et al., 2012), enabling repeated high-quality imaging and deeper visualization in scattering tissues with better stability.

Furthermore, successful intravital imaging relies on stable fluorescent labeling strategies. These include fluorescent dyes such as Carboxyfluorescein succinimidyl ester (CFSE) and Chloromethylbenzamido tetramethylrhodamine (CMTPX), as well as knock-in or transgenic mouse models expressing fluorescent proteins (Qi et al., 2016; Xu et al., 2012; Abe and Fujimori, 2013; Fink et al., 2010; Faust et al., 2000). However, these labeling strategies require careful consideration of fluorescence dilution, photobleaching, and potential physiological perturbations. In oncovirus research, additional complexities come from virus infection and trafficking mechanisms beyond conventional TME formation. To trace from viral infection to TME formation, labeling viruses and infected cells is necessary. Recent studies suggest strategies to label infected cells, enabling dynamic visualization of viral infection, TME remodeling, and immune surveillance *in vivo* using intravital imaging (Yilmaz et al., 2022).

While intravital imaging enables quantitative analysis of cellular behaviors such as migration speed and spatial proximity, it risks remaining purely descriptive. Combining it with molecular methods such as single-cell sequencing, spatial transcriptomics, mass cytometry, and genetic analysis is crucial for linking behaviors to differentiation, exhaustion, antigen presentation, and stromal interactions. These multimodal approaches enable mechanistic interpretation from imaging data (Li et al., 2025; Deng et al., 2024; Elaimy et al., 2019; Bendall et al., 2011; Haase et al., 2022).

In summary, significant challenges remain in understanding oncovirus-driven TME formation and developing therapies. However, new animal models, window platforms, and molecular technologies are expanding the experimental possibilities for studying TME dynamics. As oncovirus research adopts dynamic and systems views, intravital imaging will become key to understanding how chronic infection alters tissue structure, immune responses, and therapy outcomes. Going forward, combining spatial and molecular data will be crucial for turning viral oncology insights into effective treatments.

## 5 Conclusion and future perspectives

Oncovirus-driven tumorigenesis evolves through persistent virus-host interactions that reshape the TME. These processes are dynamic, shaped by cell-cell interactions, immune cell positioning, and tissue restructuring. These characteristics make oncovirus-associated cancers compelling platforms for intravital imaging of cell-cell interactions driven by tumor antigens and dynamic responses to immunotherapy. Intravital imaging enables real-time visualization of tumor growth, immune responses, angiogenesis, and therapeutic responses within intact tissues. This approach reveals that immune evasion and therapeutic resistance arise from coordinated cellular behaviors, including immune cell migration, immune synapse formation, trafficking, and interactions within tissue niches. These observations provide new insights into the dynamic mechanisms underlying virus-associated cancers. Future studies using physiologically relevant model systems, EBV-humanized mice, and humanized liver mice (or human liver-chimeric mice) for HBV and HCV, are expected to enable real-time visualization and quantitative analysis of immune cell infiltration, immune-tumor interaction and therapeutic responses in oncovirus-driven cancers. Therefore, intravital imaging is a powerful *in vivo* approach for spatiotemporal interrogation of TME dynamics and therapeutic mechanisms in virus-associated malignancies.
